# Carbohydrate Intake and Bacterial Vaginosis: A Systematic Review

**DOI:** 10.1177/15598276251367659

**Published:** 2025-08-28

**Authors:** Marie-Hannah Baliakas, Robert Davies

**Affiliations:** 1 Faculty of Health and Life Sciences 120958 , Coventry University, Coventry, UK (MHR); 2 Clinical Education 4954 , Central and North West London NHS Foundation Trust, London, UK (RD)

**Keywords:** carbohydrates, nutrition, bacterial vaginosis, fibre, diet, women, females

## Abstract

Bacterial vaginosis (BV) is the most prevalent vaginal infection of reproductive-aged women, with unclear causes and links to adverse gynaecological health outcomes. Fluctuations in gut microbiota from dietary carbohydrate intake may alter the vaginal microbiota. This systematic review aimed to identify an association between BV and dietary carbohydrate intake, Glycaemic index (GI) and Glycaemic Load (GL), total sugars, and dietary fibre. A literature search was conducted in April 2022 using MEDLINE, CINAHL, and Scopus databases. The pre-determined inclusion criteria were females, nutritional intake, diet, macronutrients, BV, and vaginal dysbiosis. The risk of bias was assessed using the AXIS tool for cross-sectional studies, and the CASP tool quality assessed the case-control. Four studies met the inclusion criteria: 3 cross-sectional and 1 case-control. The findings showed a positive association between higher GL intake and BV and an inverse association between higher dietary fibre intake and BV. However, the overall risk of bias was moderate to high. While a diet high in fibre may be protective and high GL may increase BV risk, the limited and inadequate quality evidence means these findings should be interpreted with caution. Further research is necessary to confirm these associations and inform dietary recommendations.

‘Dietary modifications may have therapeutic benefits for patients with re-occurring BV’.

## Key Points

(1) A diet higher in fibre intake may help reduce the risk of re-occurring bacterial vaginosis.
(2) A higher glycaemic load diet may be associated with an increased risk of bacterial vaginosis.
(3) Nutritional advice on diet quality (fibre intake and glycaemic load) could be a beneficial element in the prevention of re-occurring bacterial vaginosis.

## Introduction

Bacterial vaginosis (BV) is a condition in which there is dysbiosis in the vaginal microbiota,^1^ with BV being the most prevalent vaginal infection in women of reproductive age.^2^ The prevalence in the UK is between 10%–20%^1^ and based on the NHANES 2001-2004^3^ study, 29.2% of women between the ages of 14-49 were affected and 84% reported to be asymptomatic. Additionally, BV is more prevalent among women of colour, however there is limited understanding of the cause.^4^

BV is not a sexually transmitted disease (STI); however, it can be transmitted through intercourse^5^ and is known for being highly persistent even after treatment^6,7^ with no clear aetiology. Risk factors include vaginal douching,^8^ having new or multiple partners,^9^ smoking, using a copper intrauterine device, menstruation, and semen in the vagina.^10^

BV has been associated with serious adverse health outcomes. These include increased risk of infertility^11,12^; adverse pregnancy outcomes^13^; pelvic inflammatory disease (PID)^14^; endometriosis^15^; human immunodeficiency virus (HIV)^16^ and STIs including chlamydia, gonorrhoea^17^ and human papillomavirus (HPV).^18^ Additionally, symptoms, such as a fishy odour,^8^ have caused feelings of embarrassment for women and has negatively impacted their self-esteem and sex life.^19^

Even though it is diagnosed in millions of women every year, BV continues to be a condition that is ill-defined, unclearly diagnosed, and poorly treated.^20^

### Gut Microbiota & BV

There is emerging evidence suggesting an important relationship between nutrition and the gut microbiota, with diet having an important role in mediating the microbial system of the gastrointestinal tract.^21^ Scott et al.^22^ suggest that altering the amount and/or type of dietary carbohydrate can have a serious impact on the composition of the gut. Additionally, Bernalier-Donadille^23^ indicates that the available substrates for gut fermentation, such as plant polysaccharides, are correlated with the metabolic functions of the human gut microbiota affecting overall health.

A potential explanation for the association between carbohydrate and BV is the microbial system of the gastrointestinal tract being linked to the vaginal microbiota. Antonio et al.,^24^ found similarities between *Lactobacillus bacteria* species in the vagina and rectum, suggesting that the gut microbiota could serve as a source for vaginal colonisation via the rectum. This is also supported by Marrazzo et al.^25^ who found that non-BV women may acquire BV via extravaginal colonisation of BV associated bacteria. This suggests that alternations to the gut microbiota via dietary carbohydrate may cause changes to the vaginal microbiota causing BV. Although this may provide a temporary explanation of the relationship between the gut and vaginal microbiota, evidence is still limited, and more research is needed.

An article review by Mizigier et al.^26^ examined the role of diet and probiotics in the prevention and treatment of BV in non-pregnant women and adolescent girls. Regarding the influence of dietary carbohydrate on BV, it was stated that the development of abnormal vaginal flora was promoted by the dietary intake of simple sugars, and that maintaining a low dietary glycaemic load would benefit the vaginal area by preventing pathogenic microorganisms becoming established and reducing the inflammatory response. It was also suggested that as prebiotics, which can include types of dietary fibre, can benefit the gut microbiome and overall health, they may be able to promote vaginal health; however, the review did not provide specific details on the link between prebiotics and BV or vaginal health more widely.

In a literature review by Barrientos-Duran et al.,^27^ modifiable and non-modifiable factors that could affect vaginal microbiota homeostasis were considered which included probiotics and diet. The discussion relating to diet was predominately based on BV studies that found nutrients such as Beta-carotene, vitamins (A, B, C, and E) and minerals (zinc and calcium) connected to the reduction of BV prevalence and overall vaginal wellness. However, GL was referred to with a higher GL being related to BV progression and persistence, but no further information on dietary carbohydrate intake was discussed. Furthermore, the use of prebiotics, and dietary fibre, and how these may influence BV was not discussed.

To the best of the researchers’ current knowledge, there is no systematic review assessing the association between dietary carbohydrate and BV. The aim of this review is to identify if there is an association between BV and dietary carbohydrate intake, including GI and GL, total sugars, and dietary fibre.

## Methods

This review was conducted and reported using the PRISMA 2020 checklist^28^ to support transparency in reporting.^29^

### Eligibility Criteria

The inclusion and exclusion criteria (Table 1) included females, diet, macronutrients, nutritional intake, BV, and vaginal dysbiosis, while the study design included ethically approved primary research on human subjects. Any studies that included pregnant women were also excluded due to the fluctuation in vaginal microbiota that is caused during pregnancy^30^ which could affect BV prevalence.

**Table 1.** Inclusion and Exclusion Criteria.

	Inclusion Criteria	Exclusion Criteria
Population	Females	Males
Exposure	Nutritional intake, diet, macronutrients	Any other factors/lifestyle behaviours, anything inserted intravaginally, probiotic supplements, micronutrients, nutritional supplements
Outcome	Incidence of bacterial vaginosis (BV), vaginal dysbiosis	Any other vaginal related diseases/infections
Study design	Primary research, human, ethically approved	Secondary research, qualitative studies, in vitro

### Search Strategy

A full search strategy was developed by testing and refining it to achieve relevant studies (Supplement 1). MEDLINE, CINAHL, and Scopus health care databases were used for the search in April 2022. Limiters used were Human, English language and Females and to ensure that no studies were missed, the timeframe was not limited to the publication date. Lastly, after the title and abstract were screened, the reference lists of the studies were also searched for additional relevant studies.

### Data Extraction

The data extraction tool used was created for this review by piloting it on an included study and adapting it accordingly to ensure its usability^31^ (Supplement 2). The adaptation primarily focused on retrieving the appropriate details of assessment methods and specific results. The data extracted included study and participant characteristics, assessment methods for BV and dietary exposure, and results relating to BV comparison, dietary carbohydrate intake, GI and GL, total sugars, and dietary fibre. They were grouped into GI&GL and carbohydrates, total sugars and fibre for synthesis. Only one reviewer screened each record and summary tables were used to display data.

### Risk of Bias Assessment

The risk of bias for included studies was assessed using the AXIS tool^32^ for cross-sectional studies. The case-control was not assessed for risk of bias, it was only quality assessed with the CASP tool.^33^

## Results

### Study Selection

**Figure 1.**
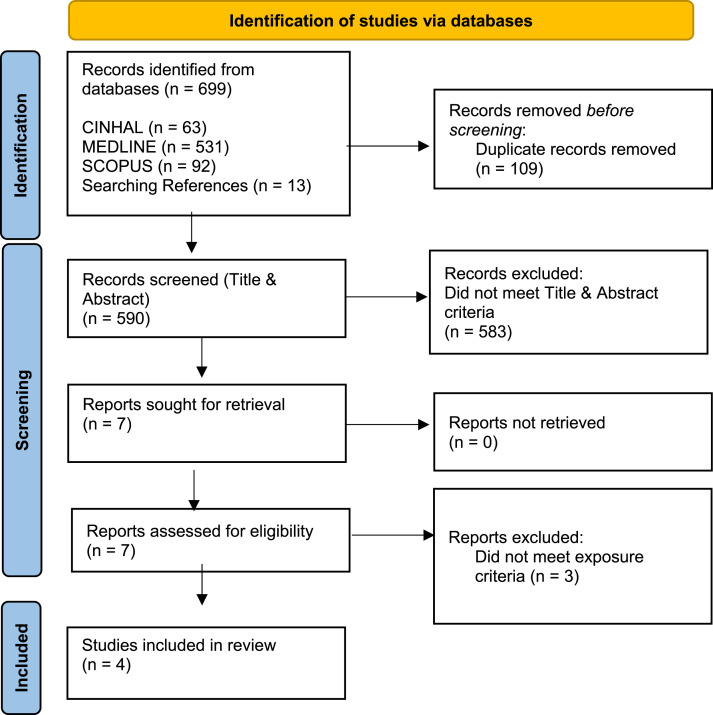
Prisma flow chart.

Once duplicates were removed, the titles and abstracts of 590 studies were screened against the inclusion and exclusion criteria, with 7 studies remaining (Figure 1). They were sought for retrieval and full text screened; 3 did not assess BV, so they were excluded, and 4 eligible studies remained.^34-37^ Only one reviewer screened each record.

### Study Characteristics

Of the included 4 studies (Table 2), 3 were cross-sectional and 1 case-control, no RCTs were found. Three were conducted in the United States (US) and one in Iran; the average duration was 12 months. An overview of the exposure and outcome assessments used in each study can be found in Supplement 3.

**Table 2.** Study Characteristics.

Author(s) & Year of Publication	Design	Setting and Country	Final sample size (n = )	Study Duration	Participant Eligibility Assessment
Neggers et al (2007)	Observational Cross-sectional & prospective analyses (sub-study to LSVF)	Routine care at health clinics Birmingham Alabama, USA	n = 1521	August 1999 – February 2002	Assessed at baseline and followed quarterly for 1 year (pelvic exam at each visit & FFQ at 2^nd^ visit) Total 5 visits
Noormohammadi et al (2022)	Observational Retrospective Case-control	Hospital-based gynaecology clinic Imam Hossein Hospital, Tehran, Iran	Cases: n = 144 Control: n = 151	November 2020 – June 2021	Convenience sampling of patients diagnosed with BV within previous 3 months & Dietary data collected of intake of past year before BV diagnosis for cases and one year before the interview for controls
Shivakoti et al (2020)	Observational Cross-sectional analysis (sub-study to HCLS)	Baseline visit of HCLS Baltimore, USA	n = 104	Recruited 2011-2015	Assessed at baseline visit with mid-vaginal E-swabs & FFQ questionnaire
Thoma et al (2011)	Observational Cross-sectional & prospective analyses (sub-study to LSVF)	Routine care at health clinics Birmingham Alabama, USA	n = 1735	August 1999 – February 2002	Assessed at baseline and followed quarterly for 1 year (pelvic exam at each visit & FFQ at 2^nd^ visit) Total 5 visits

Key LSVF: Longitudinal Study of Vaginal Flora; HCLS: Hormonal Contraception longitudinal Study; FFQ: Food Frequency Questionnaire; USA: United States of America; n: number

### Participant Characteristics

The mean age of participants ranged from 25 (±6.7) to 31.44 (±7.55) years and reported race was African American (67.1%), Caucasian (28.3%) and (12%) other, however Noormohammadi et al.^34^ did not report race. Two studies reported BV prevalence with the mean being 27.23%, while the other two reported BV cases in numbers (Table 3).

**Table 3.** Participant Characteristics.

Author(s) & year of publication	Inclusion Criteria	Mean age (y) (±SD)	Race (%)	BV prevalence (%) or Number of BV cases
Neggers et al (2007)	Healthy non-pregnant 15-44 years	25 ± 6.7 y	86% African American 14% Caucasian	Prevalence: BV 41.8% Severe BV: 14.9%
Noormohammadi et al (2022)	18-45 years Cases: Women diagnosed with BV Controls: Women with no ongoing or previous BV or BV treatment	Cases: 30.10 ± 6.03 y Controls: 31.44 ± 7.55 y	NR	BV cases: 144 Non-BV control: 151
Shivakoti et al (2020)	Reproductive-aged women who were using HC or reported planning to cease or initiate HC or were not using HC	25.9 y ± SD NR	58% Caucasian 30% Black 12% Other	Prevalence: BV 25% at entry
Thoma et al (2011)	Healthy non-pregnant 15-44 years	25.3 ± 6.8 y	85.5% African American 14.5% Caucasian	Normal flora = 598 Intermediate flora = 379 BV = 742

Key BV: Bacterial Vaginosis; BMI: Body Mass Index; NR: Not reported; SD: Standard deviation; HC: Hormonal contraceptive

### Nutrient Results

The results were separated into GI & GL (Table 4) and carbohydrate (including total sugars and fibre) (Table 5). The studies were split based on the two groups. Thoma et al.^35^ did not report some *P*-values and thus an approximation was calculated using the *P*-value calculation formula.^38^

**Table 4.** GI & GL Results.

Thoma et et al (2011)												
	Cross-Sectional Analysis				Prospective Analysis							
	BV vs Normal Flora				BV Progression vs non-BV Maintenance				BV Persistence vs non-BV Maintenance			
	cOR (95% CI)	*P* value	aOR^a^ (95% CI)	*P* value	cOR (95% CI)	*P* value	aOR^a^ (95% CI)	P value	cOR (95% CI)	*P* value	aOR^a^ (95% CI)	*P* value
GI	0.92 (0.68-1.25)	NR	0.99 (0.71-1.38)	NR	0.98 (0.60-1.61)	NR	1.03 (0.61-1.75)	NR	0.82 (0.58-1.16)	NR	0.99 (0.67-1.46)	NR
GL	1.02 (1.01-1.03)	<0.001^b^	1.01 (1.00-1.03)	0.037	1.03 (1.01-1.05)	0.0028^b^	1.03 (1.00-1.05)	0.018^b^	1.02 (1.01-1.04)	0.008^b^	1.02 (1.01-1.04)	0.008^b^

Noormohammadi et al (2022)												
	BV vs Control – Carbohydrate indices mean intake			Tertile carbohydrate indices & BV odds								
				1^st^		2^nd^		3^rd^		P value for trend		
	BV	Control	P value	aOR^c^ (95% CI)	aOR^d^ (95% CI)	aOR^c^ (95% CI)	aOR^d^ (95% CI)	aOR^c^ (95% CI)	aOR^d^ (95% CI)	aOR^c^	aOR^d^	
GI	61.24	59.49	<0.001	1.00 (ref.)	1.00 (ref.)	2.10 (1.12-3.95)	2.10 (1.02-4.31)	2.95 (1.59-5.46)	2.99 (1.47-6.08)	0.001	0.003	
GL	190.8	162.0	0.069	1.00 (ref.)	1.00 (ref.)	0.85 (0.47-1.53)	1.12 (0.50-2.49)	1.55 (0.90-2.69)	4.01 (1.22-5.91)	0.102	0.029	

Shivakoti et Al (2020)												
Cross-Sectional Analysis												
	Association of Dietary Intake & Molecular-BV											
	aOR^e^ (95% CI)	P value	aOR^f^ (95% CI)	P value								
GI	0.99 (0.86-1.13)	0.93	0.99 (0.86-1.13)	0.87								
GL	1.63 (0.56-4.74)	0.37	0.72 (0.21-2.55)	0.62								

Key BV: Bacterial Vaginosis; GI: Glycaemic Index; GL: Glycaemic Load; BMI: Body Mass Index; WC: Waist Circumference; CI: Confidence Interval; NR: Not reported

^a^: Adjusted for age, race, education, cigarette smoking, alcohol use, BMI, douching frequency, hormonal contraceptive use, and number of sex partners

^b^: Calculated using *P*-value formula

^c^: Adjusted for age

^d^: Adjusted for age, BMI (kg/m^2^), WC (cm), cigarette smoker, energy intake (kcal/d), fat intake (g/d), familial history of BV and physical activity (MET/h/d)

^e^: Adjusted for total energy intake

^f^: Adjusted for total, energy, BMI, and hormonal contraception use

**Table 5.** Carbohydrate, Total Sugars & Fibre Results.

Noormohammadi et al (2022)											
Macronutrients	BV vs Control – Macronutrient mean intake			Tertile carbohydrate indices & BV odds							
				1^st^		2^nd^		3^rd^		P value for trend	
	BV	Control	P value	aOR^a^ (95% CI)	aOR^b^ (95% CI)	aOR^a^ (95% CI)	aOR^b^ (95% CI)	aOR^a^ (95% CI)	aOR^b^ (95% CI)	aOR^a^	aOR^b^
Carbohydrate	325.7 (g/d)	307.5 (g/d)	0.463	1.00 (ref.)	1.00 (ref.)	0.93 (0.53-1.66)	1.03 (0.45-2.37)	1.16 (0.66-2.03)	1.46 (0.37-5.86)	0.594	0.640
Total sugars	N/A	N/A	N/A	N/A	N/A	N/A	N/A	N/A	N/A	N/A	N/A
Fibre	20.1 (g/d)	23.8 (g/d)	0.013	1.00 (ref.)	1.00 (ref.)	1.24 (0.73-2.11)	0.72 (0.36-1.45)	0.36 (0.19-0.69)	0.22 (0.14-0.33)	0.007	<0.001

Neggers et al (2007)											
Cross-sectional analysis											
Macronutrients	Macronutrient quartiles & BV					Macronutrient quartiles & severe BV					
	1^st^	2^nd^	3^rd^	4^th^	P value Trend	1^st^	2^nd^	3^rd^	4^th^	P value Trend	
	aOR (95% CI)	aOR (95% CI)	aOR (95% CI)	aOR (95% CI)		aOR (95% CI)	aOR (95% CI)	aOR (95% CI)	aOR (95% CI)		
Carbohydrate^b^	(Referent)	1.0 (0.8-1.4)	1.1 (0.8-1.5)	1.1 (0.8-1.5)	0.41	(Referent)	0.9 (0.6-1.4)	0.9 (0.6-1.4)	0.8 (0.5-1.3)	0.86	
Total sugars	N/A	N/A	N/A	N/A	N/A	N/A	N/A	N/A	N/A	N/A	
Fibre	DNS	DNS	DNS	DNS	DNS	DNS	DNS	DNS	DNS	DNS	

Shivakoti et al (2020)											
Macronutrients	Cross-sectional analysis										
	Association of dietary intake & molecular-BV										
	aOR^c^ (95% CI)			P value			aOR^d^ (95% CI)			P value	
Carbohydrate	1.24 (0.45-3.41			0.68			0.64 (0.18-2.23)			0.48	
Total sugars	1.36 (0.71-2.59)			0.35			1.01 (0.49-2.11)			0.97	
Fibre	0.45 (0.23-0.87)			0.02			0.49 (0.24-0.99)			0.049	

Key BV: Bacterial Vaginosis; CI: Confidence Intervals; N/A: Not Assessed; BMI: Body Mass Index; DNS: Data not Shown; WC: Waist circumference

^a^: Adjusted for age

^b^: Adjusted for age, BMI (kg/m^2^), WC (cm), cigarette smoker, energy intake (kcal/d), fat intake (g/d), familial history of BV and physical activity (MET/h/d)

^c^: Adjusted for total energy intake

^d^: Adjusted for total, energy, BMI, and hormonal contraception use

^e^: Adjusted for protein, carbohydrate, age, race, BMI (BV only), income, education (BV only), douching, contraceptive pill use, and alcohol intake (BV only)

### GI & GL

Noormohammadi et al.^34^ compared the mean intake of GI and GL between BV cases and controls, the only statistically significant result was that BV cases were associated with a higher GI diet (*P* < 0.001). Carbohydrate tertile indices and their association with BV odds were also evaluated, and results adjusting for energy intake showed a significant association with BV in the higher GI tertile compared to lower (adjusted Odds Ratio (aOR) 2.95, Confidence Interval (CI) (1.59-5.46), *P* = 0.001) and after adjusting for other factors the results remained statistically significant (aOR 2.99, CI 1.47-6.08, *P* = 0.003). GL after adjusting for other factors was also statistically significant in the higher tertile (aOR 4.01, CI 1.22-5.92, *P* = 0.029).

Thoma et al.^35^ in the cross-sectional analysis compared the odds of intermediate or BV flora to normal flora per 10-unit increase in GI and GL. For the crude results, although *P*-values were not reported, higher GL was positively associated with BV compared to normal flora (crudes Odds Ratio (cOR 1.02, CI 1.01-1.03. *P*≈<0.001). This approximation was calculated using the CI 1.01-1.03 and cOR 1.02 and the *P*-value calculation formula.^38^ For adjusted results, higher GL was also positively associated with BV compared to normal flora (aOR 1.01, CI 1.00-1.03, *P* = 0.037).

In the prospective analysis the association between GI and GL with BV progression, persistence and resolution over time was examined. For crude results the *P* values were not reported, and GI results were not statistically significant; however, higher GL was positively associated with BV progression (cOR 1.03, CI 1.01-1.05, *P*≈0.0028) as well as persistence (cOR 1.02, CI 1.01-1.04, *P*≈0.008). For adjusted results, GI results were not statistically significant, however, for higher GL there was a positive association with BV progression (aOR 1.03, CI 1.00-1.05, *P*≈0.018) and persistence (aOR 1.02, CI 1.01-1.04, *P*≈0.008). All approximate *P-*values in the prospective analysis were calculated using the same formula.^38^ The results from Shivakoti et al.^36^ had no statistical significance.

### Carbohydrate, Total Sugars and Fibre

Noormohammadi et al.^34^ compared the mean intake of carbohydrate and fibre intake in BV cases vs controls. Statistical significance was associated with a higher fibre intake in controls (*P* = 0.013). After adjusting for energy intake, the analysis of carbohydrate tertiles and BV odds revealed a statistically significant association, with lower odds of BV observed in the highest fibre tertile (aOR 0.36, CI 0.19-0.69, *P* = 0.007) and still after further adjustments (aOR 0.22, CI 0.14-0.33, *P*=<0.001). No other results were statistically significant.

Shivakoti et al.^36^ examined carbohydrate, total sugars, and fibre intake with BV odds. In results adjusted for energy intake, a higher total fibre intake was inversely (aOR 0.45, CI 0.23-0.87, *P* = 0.02) associated with BV. For further adjustments a higher total fibre intake was still inversely associated (aOR 0.49, CI 0.24-0.99, *P* = 0.049). No other results were statistically significant.

Neggers et al.^37^ looked at BV in relation to carbohydrate and fibre trends. The carbohydrate results were not statistically significant, and the fibre results were not reported.

### Risk of Bias

The quality of the studies was varied. Thoma et al.^35^ and Neggers et al.^37^ were found to have a high risk of bias and Shivakoti et al.^36^ was found to have some concerns using the AXIS tool (Supplement 4). For Noormohammadi et al.,^34^ only a quality assessment was completed using the CASP tool (Supplement 5).

The studies did not justify their sample size nor was it clear how participants were selected, even though the sample was from an appropriate population, potentially causing selection bias.^39^

All the cross-sectional studies^35-37^ had concerns with reporting their data; basic data often was not presented or only briefly mentioned potentially leading to missing data bias^40^; only Shivakoti et al.^36^ presented all the data that was analysed and performed a sensitivity analysis.

## Discussion

This review aimed to identify whether there is an association between dietary carbohydrate and BV. Overall, the results suggest that a higher GL diet may be positively associated with BV and a diet high in fibre may have an inverse effect on BV.

Noormohammadi et al.^34^ found that the higher tertile of GL intake was positively associated with BV odds in adjusted results and Thoma et al.^35^ supported this as participants with BV (odds, progression, persistence) were positively associated with a higher GL diet (Table 4). However, Shivakoti et al.^36^ did not find this association, but the evidence could suggest an association between GL and BV.

There are two difference types of dietary carbohydrate: digestible and non-digestible. Digestible carbohydrate are comprised of starches and sugars, such glucose, fructose, sucrose, and lactose.^21^ The amount and type of carbohydrate in a meal can influence someone’s glycaemic response.^41^ The GL score indicates how fast glucose enters the bloodstream of a particular food as well as how much glucose per serving is supplied; it gives a more accurate picture of how the dietary carbohydrate impacts blood sugar levels.^42^ When glucose stimulates insulin release, it has been found to potentially cause oxidative damage leading to increased inflammation, due to continuous exposure to hyperglycaemia.^43-45^ As the inflammation can affect the gut microbiota^46^; it is possible that constant exposure to high-GL foods affects bacterial colonisation in the vagina due to impaired immune response.^24,25^ Additionally, studies have found that digesting high levels of glucose, fructose, and sucrose increased the abundance of *Bifidobacteria*, and decreased *Bacteroides* in the gut, possibly altering both microbiomes.^47,48^ However, these are theories and do not directly justify the association.

Noormohammadi et al.^34^ found a higher fibre intake in controls than in BV cases (Table 5). In the carbohydrate tertile analysis, lower BV odds were associated with high fibre intake for both adjusted models (Table 5). Shivakoti et al.^36^ supports this as the cross-sectional analysis found an inverse association between fibre intake and BV odds in both adjusted models (Table 5). Neggers et al.^37^ assessed fibre but did not report the results.

This association may also be related to the gut and vaginal microbiota. Fibre is the non-digestible type of dietary carbohydrate, which is metabolised by specific species of gut microbiota via anaerobic fermentation.^49^ Dietary fibre has a vital effect on the diversity and composition of the microbiome, providing fermentation opportunities.^49^ It has been found to modulate the gut towards a healthier composition,^49-51^ which may, in turn, affect bacterial colonisation in the vagina towards a *Lactobacillus*-dominated composition via the rectum.

Consuming higher amounts of dietary fibre has the potential to alter the nutritional function of the intestine which promotes the growth of beneficial gut bacteria.^52^ Noormohammadi et al.^34^ found that women with BV had an average intake of 20.1 g/day and the control had 23.8 g/day, a difference of 3.7 g/day, while Shivakoti et al.^36^ found the median intake of fibre was 9.6 g/day per 1000 kcal/day. The recommended fibre intake for an adult in the UK is 30 g/day,^53^ and currently adults in the UK between 19 to 64 years have a mean intake of 19.7 g a day.^54^

Confounding factors should also be considered.^55^ Hormonal contraception (HC) can influence BV occurrence, as both progesterone and oestrogen can cause different mechanisms that can increase the posibilities of vaginal dysbiosis.^56^ Therefore, it is important that HC was adjusted for as it could affect the outcome of the results. Shivakoti et al,^36^ Neggers et al^37^ and Thoma et al.^35^ adjusted for HC, however, Noormohammadi et al.^34^ did not, resulting in possible errors in outcome measurements.

## Strengths and Limitations

A strength of this review is that it sought to investigate the association between dietary carbohydrate intake and BV which is a topic that has received little attention in the field of women’s health. It also highlights the need for further research concerning women’s health and the influence of dietary intake on women’s sexual health. The review utilised a comprehensive database search with clear inclusion criteria (Table 1) to ensure relevant studies were identified. To enhance its transparency, the CRD^57^ guidance for reporting methodology was used as well as the PRISMA 2020^28^ checklist.

Limitations of the review included limiting the search to studies reported in English causing language bias.^58^ Furthermore, a limited number of studies were found which hindered interpretation, synthesis and analyses of the results. Only one reviewer screened records and collected data putting the review at risk of selection bias.^39^

## Conclusion and Future Research Recommendations

The results suggest that a higher GL diet may have a positive association with BV incidence, there is an inverse association with BV and a diet higher in fibre. However, the included studies were of moderate to poor quality, so even though the results are interesting they need to be interpreted with caution.

Based on the results of the present review, specific dietary recommendations cannot be made. However, as discussed, the gut microbiome composition is significantly affected by dietary changes, and potentially the vaginal microbiome. Specifically, some studies^21-23^ show that dietary fibre has a positive effect on the gut microbiome, and Noormohammadi et al.^34^ found that the control group had a 3-4 g/day higher fibre intake than the BV group. Therefore, women should be advised to monitor their dietary carbohydrate intake specifically focusing on dietary fibre and aiming to achieve current dietary recommendations.

Dietary modifications may have therapeutic benefits for patients with re-occurring BV. Given the sparse research into dietary intake and its relation to women’s gynaecological health, future research is recommended.

## Supplemental Material

Supplemental material - Carbohydrate Intake and Bacterial Vaginosis: A Systematic Review Supplemental material for Carbohydrate Intake and Bacterial Vaginosis: A Systematic Review by Marie-Hannah Baliakas and Robert Davies in American Journal of Lifestyle Medicine

Supplemental material - Carbohydrate Intake and Bacterial Vaginosis: A Systematic Review Supplemental material for Carbohydrate Intake and Bacterial Vaginosis: A Systematic Review by Marie-Hannah Baliakas and Robert Davies in American Journal of Lifestyle Medicine

Supplemental material - Carbohydrate Intake and Bacterial Vaginosis: A Systematic Review Supplemental material for Carbohydrate Intake and Bacterial Vaginosis: A Systematic Review by Marie-Hannah Baliakas and Robert Davies in American Journal of Lifestyle Medicine

Supplemental material - Carbohydrate Intake and Bacterial Vaginosis: A Systematic Review Supplemental material for Carbohydrate Intake and Bacterial Vaginosis: A Systematic Review by Marie-Hannah Baliakas and Robert Davies in American Journal of Lifestyle Medicine

Supplemental material - Carbohydrate Intake and Bacterial Vaginosis: A Systematic Review Supplemental material for Carbohydrate Intake and Bacterial Vaginosis: A Systematic Review by Marie-Hannah Baliakas and Robert Davies in American Journal of Lifestyle Medicine

## Data Availability

The authors confirm that all data generated or analysed during this study are included in this published article.*
