# Supplementary material for: Carbohydrate Intake and Bacterial Vaginosis: A Systematic Review
Source: Am J Lifestyle Med. 2025 Aug 28:15598276251367659. Online ahead of print. doi: 10.1177/15598276251367659 (PMC12394200; doi:10.1177/15598276251367659)
Supplement: Supplemental material - Carbohydrate Intake and Bacterial Vaginosis: A Systematic Review [file sj-pdf-1-ajl-10.1177_15598276251367659.pdf]

Supplement 1 - Search Strategy

POPULATION		EXPOSURE		OUTCOME
Search Terms				
Female*<br>OR<br><br>Synonym: Women OR Woman<br>OR<br><br>MeSH term or subject heading as Major Concept (MM ‘Women’)	AND	Nutritional Intake<br>OR<br><br>Synonym: Diet* OR Nutrition* OR Nutrient*<br>OR<br><br>MeSH term or subject heading as Major Concept (MM ‘Diet’, MM ‘Nutrients’)	AND	Bacterial vaginosis<br>OR<br><br>Synonym: Vagin* OR Micro*<br><br>OR<br><br>MESH term subject heading as Major Concept<br>(MM ‘Vaginosis, Bacterial’)
