# Supplementary material for: Carbohydrate Intake and Bacterial Vaginosis: A Systematic Review
Source: Am J Lifestyle Med. 2025 Aug 28:15598276251367659. Online ahead of print. doi: 10.1177/15598276251367659 (PMC12394200; doi:10.1177/15598276251367659)
Supplement: Supplemental material - Carbohydrate Intake and Bacterial Vaginosis: A Systematic Review [file sj-pdf-2-ajl-10.1177_15598276251367659.pdf]

Supplement 2– Data Extraction Tool

General Information		Details	
	Title of study		
	Author (s)		
	Year of publication		
	Country		
	Reference citation		
Methods		Details	
	Design		
	Aims/Objectives		
	Setting		
	Duration		
	Participation Assessment		
	Ethical approval		
Participants		Details	
	Inclusion criteria		
	Sample size		
	Mean Age		
	Race		
	Mean BMI		
	BV prevalence		
Assessment Method Overview		Details	
	Exposure		
	Outcome		
	Vaginal flora or BV categorization		
	Vaginal flora or BV evaluation		
Results		Details	
	Results looking at GI & GL		
	Type of analysis	i.e., Cross-sectional analysis	
	BV comparison	i.e., BV progression	
	Results	i.e., OR, aOR, CI, p value	
	Results looking at Carbohydrates, Total Sugars& Fibre		
	Type of analysis	i.e., Cross-sectional analysis	
	BV comparison	i.e., BV progression	
	Results	i.e., OR, aOR, CI, p value	

Key<br>BMI: Body Mass Index<br>BV: Bacterial Vaginosis<br>GI: Glycaemic Index<br>GL: Glycaemic Load	OR: Odds Ratio<br>aOR: adjusted Odds Ratio<br>CI: Confidence Interval
