# Supplementary material for: Carbohydrate Intake and Bacterial Vaginosis: A Systematic Review
Source: Am J Lifestyle Med. 2025 Aug 28:15598276251367659. Online ahead of print. doi: 10.1177/15598276251367659 (PMC12394200; doi:10.1177/15598276251367659)
Supplement: Supplemental material - Carbohydrate Intake and Bacterial Vaginosis: A Systematic Review [file sj-pdf-3-ajl-10.1177_15598276251367659.pdf]

Supplement 3 – Assessment Overview

Author(s) & year of publication	Objective	Exposure	Outcome	Vaginal Flora or BV Categorization	Vaginal Flora or BV Evaluation
Neggers et al. (2007)	To assess the relationship between dietary intake (energy and nutrients) and the presence of BV in a sample of non-pregnant women.	100-Block 98 FFQ used to collect dietary intake<br><br>Nutrients assessed:<br>Total Energy<br>Total Fat (Saturated Fat, Polyunsaturated Fat, Monosaturated Fat)<br>Protein<br>Carbohydrate<br>Micronutrients	Cross sectional analysis: Assessed relation of each energy and nutrient variable to BV and severe BV<br><br>Prospective analysis: Assessed the relationship of dietary components with maintaining BV negative vs. incident BV & persistent BV vs. remitting BV	Non-BV: Nugent score <7<br><br>BV: Nugent score ≥ 7<br><br>BV severe: Nugent score ≥ 9<br>&<br>Vaginal pH ≥ 5	Nugent Gram-strain criteria
Noormohammadi et al. (2022)	To examine the association of dietary glycaemic index (DGI), glycaemic load (DGL), insulin index (DII), and insulin load (DIL) with BV among Iranian women.	168-item Semiquantitative FFQ used to collect dietary intake<br><br>Nutrients assessed:<br>Energy intake<br>Carbohydrate & Fiber<br>Protein<br>Total fat<br>GI &GL<br>II & IL	Mean dietary intake & carbohydrate indices of cases vs. control<br>&<br>Tertile value categorization: Association between dietary carbohydrate indices & BV odds cases vs. control	BV: 3 out 4 symptoms<br>1) Homogenous & dilute vaginal discharge<br>2) Vaginal pH > 4.5<br>3) Presence of 20% clue cells<br>4) Fish odour after adding 10% potassium hydroxide to discharge slide	Amsel criteria
Shivakoti et al. (2020)	To evaluate the relationship between dietary macronutrient intake and molecular-BV	Block Brief 2000 FFQ (68 items) to assess usual dietary intake<br><br>Nutrients assessed:<br>Total Energy<br>Carbohydrate & Fiber	Cross-sectional analysis: Association between aspects of dietary intake and molecular-BV	Molecular-BV:<br>CST IV (Anaerobes including Gardnerella vaginalis, Atopobium vaginae and BVAB1),<br>CST VI (Streptococcus),<br>CST VII (Bifidobacterium)	16S rRNA gene sequencing to assign CST

		Total Fat (Saturated, Monosaturated, Polysaturated, Cholesterol, Trans Fat)<br>Protein<br>Total sugars<br>GI & GL		Non-BV:<br>CST I (L.crispatus), CST II (L.gasseri), CST III (L.iners) and CST V (L.jensenii)	
Thoma et al. (2011)	To examine the association between BV and several dietary indices among participants in the LVSF	110-Block98 FFQ used to collect dietary intake<br><br>Dietary Indices Assessed:<br>GI & GL<br>HEI score<br>NNR Score	Cross-sectional analyses:<br>Association between dietary indices and vaginal flora (Intermediate & BV)<br><br>Prospective analyses:<br>Association of dietary indices with BV progression, resolution, and persistence compared to maintenance of non-BV<br>&<br>BV resolution compared to persistence	Cross-sectional analyses:<br>Vaginal Flora Assessment<br>Normal: Nugent score 0-3<br>Intermediated: Nugent score 4-6<br>BV: Nugent score 7-10<br><br>Prospective analyses:<br>BV Quartiles<br>1) BV negative at both visits (non-BV maintenance)<br>2) BV negative at the current visit and positive at next (BV progression)<br>3) BV positive at the current visit and negative at the next (BV resolution)<br>4) BV positive at both visits (BV persistence)	Nugent Gram-strain criteria

<u>Key</u><br>BV: Bacterial Vaginosis<br>CST: Community State Type<br>GI: Glycaemic Index<br>GL: Glycaemic Load	HEI: Healthy Eating Index<br>NNR score: Naturally Nutrient Rich score<br>II: Insulin Index<br>IL: Insulin Load<br>FFQ: Food Frequency Questionnaire
