# Supplementary material for: Carbohydrate Intake and Bacterial Vaginosis: A Systematic Review
Source: Am J Lifestyle Med. 2025 Aug 28:15598276251367659. Online ahead of print. doi: 10.1177/15598276251367659 (PMC12394200; doi:10.1177/15598276251367659)
Supplement: Supplemental material - Carbohydrate Intake and Bacterial Vaginosis: A Systematic Review [file sj-pdf-4-ajl-10.1177_15598276251367659.pdf]

Supplement 4 - Cross-sectional Bias Risk Assessment

Questions	Neggers et al. (2007)	Shivakoti et al. (2020)	Thoma et al. (2011)
Introduction			
1. Were the aims/objectives of the study clear?	Y	Y	Y
Methods			
2. Was the study design appropriate for the stated aim(s)?	Y	Y	Y
3.Was the sample size justified?	N	N	N
4. Was the target/reference population clearly defined? (Is it clear who the research was about?)	Y	Y	Y
5. Was the sample frame taken from an appropriate population base so that it closely represented the target/reference population under investigation?	Y	Y	Y
6.Was the selection process likely to select subjects/participants that were representative of the target/reference population under investigation?	Y	Y	Y
7. Were measures undertaken to address and categorise non-responders?	NC	NC	NC
8. Were the risk factor and outcome variables measured appropriate to the aims of the study?	Y	Y	Y
9. Were the risk factor and outcome variables measured correctly using instruments/measurements that had been trialled, piloted or published previously?	Y	Y	Y
10. Is it clear what was used to determined statistical significance and/or precision estimates? (e.g. p-values, confidence intervals)	Y	Y	Y
Results			
11. Were the methods (including statistical methods) sufficiently described to enable them to be repeated?	Y	Y	Y
12. Were the basic data adequately described?	N	Y	N
13*. Does the response rate raise concerns about non-response bias?	Y	Y	Y
14. If appropriate, was information about non-responders described?	N	N	N
15. Were the results internally consistent?	N	Y	N
16. Were the results presented for all the analyses described in the methods?	NC	Y	N
Discussion			
17. Were the authors' discussions and conclusions justified by the results?	Y	Y	Y
18. Were the limitations of the study discussed?	Y	Y	Y

Other			
19*. Were there any funding sources or conflicts of interest that may affect the authors' interpretation of the results?	N	N	N
20. Was ethical approval or consent of participants attained?	Y	Y	Y
Results	13/20	16/20	13/20
Risk of Bias	High risk	Some concerns	High risk

Key
Y: Yes
N: No
