# Supplementary material for: Carbohydrate Intake and Bacterial Vaginosis: A Systematic Review
Source: Am J Lifestyle Med. 2025 Aug 28:15598276251367659. Online ahead of print. doi: 10.1177/15598276251367659 (PMC12394200; doi:10.1177/15598276251367659)
Supplement: Supplemental material - Carbohydrate Intake and Bacterial Vaginosis: A Systematic Review [file sj-pdf-5-ajl-10.1177_15598276251367659.pdf]

Supplement 5 - Case-control Quality Assessment

Questions	Noormohammadi et al. (2022)
Section A: Are the results of the trial valid?	
1.Did the study address a clearly focused issue?	Yes
2.Did the authors use an appropriate method to answer their question?	Yes
3.Were the cases recruited in an acceptable way?	Not clear
4.Were the controls selected in an acceptable way?	Not clear
5.Was the exposure accurately measured to minimise bias?	Not clear
6.(a) Aside from the experimental intervention, were the groups treated equally?<br>(b) Have the authors taken account of the potential confounding factors in the design and/or in their analysis?	a. Yes<br>b. No they did not
Section B: What are the results?	
7.How large was the treatment effect?	Analysis was appropriate
8.How precise was the estimate of the treatment effect?	Not very precise
9. Do you believe the results?	Yes
Section C: Will the results help locally?	
10.Can the results be applied to the local population?	Yes
11.Do the results of this study fit with other available evidence?	Yes
Results	The quality was moderate due to the variation in results.
